# Molecular Insight into Amyloid Fibril-Templated Aggregation of Biomarkers

**DOI:** 10.1021/acschemneuro.5c00103

**Published:** 2025-05-27

**Authors:** Rongfeng Zou, Hans Ågren

**Affiliations:** † Department of Physics and Astronomy, 8097 Uppsala University, Box 516, SE-751 20 Uppsala, Sweden; ‡ Faculty of Chemistry, Wroclaw University of Science and Technology, Wyb. Wyspianskiego 27, PL-50370 Wroclaw, Poland

**Keywords:** tau fibril, molecular dynamics, ligand aggregation, neurodegenerative disorders, fibril-templated aggregation

## Abstract

The aggregation of misfolded proteins into β-sheet-rich fibrils constitutes a characteristic feature of neurodegenerative disorders and represents a therapeutic target. While cryo-electron microscopy has elucidated ordered binding patterns of small molecules on fibril surfaces, the mechanisms of ordered aggregate formation generally remain unclear. This study employs molecular dynamics (MD) simulations of the model ligand GTP-1 to examine fibril-templated ligand aggregation and elucidate the molecular determinants governing the aggregation process. Our results showed that in aqueous solution, GTP-1 molecules form dynamic clusters without preferential configurations, whereas tau fibril surfaces induce organized aggregation through protein–ligand hydrogen bonding and ligand–ligand π–π stacking interactions. 1000 independent 100 ns simulations were initiated from diverse ligand conformations to comprehensively sample the conformational landscape. Analysis of the MD trajectories revealed two distinct aggregation pathways. Starting from random initial configurations, on-pathway trajectories spontaneously sampled crystal-structure-like conformations during the simulation, and these conformations exhibited high kinetic stability after formation. In contrast, off-pathway trajectories were characterized by ligands adopting non-native binding geometries, with continuous interconversions between multiple disordered states. The conformational stability of on-pathway states was attributed to optimal surface complementarity and enhanced intermolecular interactions, while off-pathway configurations exhibited reduced structural order and increased conformational flexibility. Quantitative analysis demonstrated differential hydrogen-bonding patterns, with on-pathway aggregates forming 2.01 bonds per structure compared to 0.74 in off-pathway configurations. Energy decomposition identified protein–ligand interactions as the primary determinant of binding energetics, highlighting the direct influence of fibril surface properties on ligand aggregation. These findings provide a mechanistic basis for fibril-templated aggregation and offer a rational foundation for designing diagnostic agents targeting pathological protein fibrils in neurodegenerative diseases.

## Graphical abstract

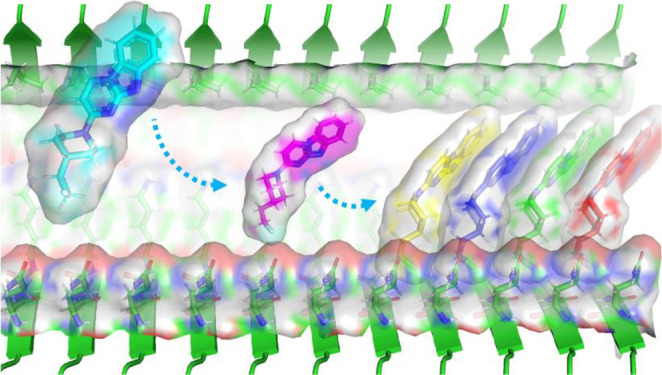

## Introduction

Neurodegenerative diseases, including Alzheimer’s disease (AD), Parkinson’s disease (PD), and Huntington’s disease (HD), represent a growing global health challenge due to their progressive and debilitating nature.^1^ These diseases are characterized by the gradual loss of neuronal function, leading to severe cognitive decline, motor dysfunction, and ultimately, an increased mortality rate.^2^ As the global population ages, the incidence of these diseases continues to rise, emphasizing the urgent need for improved diagnostic tools and therapeutic strategies.^3^ A common hallmark of these diseases is the abnormal aggregation of misfolded proteins, which form insoluble fibrillar structures.^4^ The characterization of protein–ligand interactions in these fibrils is beneficial to the development of diagnostic probes.

At the molecular level, structural studies have revealed that fibrillar proteins, including amyloid-beta (Aβ),^5^ tau,^6^ and alpha-synuclein,^7^ adopt highly ordered, β-sheet-rich architectures. These fibrils present distinct binding interfaces, which make them attractive binding sites for small-molecule binding and probe development.^8^ Advances in cryo-electron microscopy (cryo-EM) have enabled structural determination of these fibrils at near-atomic resolution, revealing their complex architectural features and potential ligand-binding sites.^9^ The binding mode of small molecules on fibril surfaces exhibits a distinct pattern characterized by ligand alignment parallel to the fibril axis in a stacked arrangement.^10^ This binding configuration is stabilized through multiple molecular interactions, including π–π stacking, hydrophobic forces, and electrostatic contributions, which collectively provide mechanistic insight into fibril-ligand recognition principles critical for probe development.^10^ The observations of stacked binding configurations across diverse molecular probes, such as GTP-1,^11^ MK-6240,^12^ and PiB,^10^ suggesting that stacked binding is a common mechanism of ligand recognition in amyloid fibril systems.

While substantial researches have been devoted to exploring individual fibril-ligand interactions, much of these works have focused on characterizing static properties, such as binding sites, affinities. For example, multiple binding sites with distinct binding affinities have been systematically identified through MD simulations and MM/GBSA calculations.^13–16^ However, these studies primarily address individual binding events, possibly leaving gaps in understanding the collective behavior of ligands on fibril surfaces.

A key unresolved question is the mechanism by which small molecules transition from individual binding events to cooperative, ordered aggregates on fibril surfaces. Structural analyses through cryo-EM have revealed features suggesting that ligand organization on fibril surfaces exhibits characteristics such as periodic binding interfaces, directional molecular interactions. These characteristics are consistent with template-assisted aggregation, which have been extensively documented in supramolecular chemistry.^17–20^ In amyloid fibrillar systems, the establishment of surface-ligand interactions enables the formation of ordered molecular aggregates, with the fibril surface functioning as a structural template. The surface architecture of fibrils, comprising hydrophobic grooves and charged domains, has been demonstrated to be crucial for ligand recognition,^21^ and could facilitate the formation of ordered aggregates (Figure 1). Although cryo-EM has provided unprecedented structural snapshots of stacked binding conformations, it cannot capture the dynamic processes that drive the formation, evolution, and stabilization of these complexes. The thermodynamic and kinetic properties underlying this cooperative behavior thus remain poorly understood. These gaps in understanding not only limit the rational design of molecular probes but also hinder efforts to explore template-assisted aggregation as a new strategy for enhancing binding specificity and affinity.

**Figure 1:**
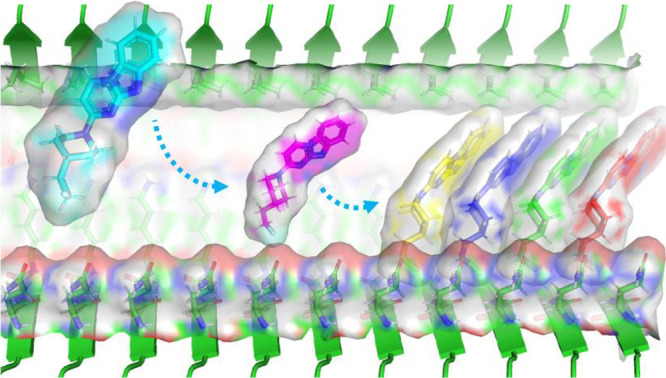
Schematic representation of fibril-templated small molecule aggregation.

In this study, molecular dynamics simulations are employed to investigate the mechanistic basis of fibril-templated ligand aggregation on tau fibril surfaces, with emphasis on the conformational dynamics and aggregation pathways of small molecules. Our simulations reveal how the fibril surface influences ligand aggregation and promotes cooperative interactions over time, thereby identifying the molecular determinants that govern binding behavior. By integrating structural observations with MD data, we gain detailed insight into the template-assisted aggregation processes and the principles underlying fibril-ligand interactions. These molecular-level findings enhance our understanding of the associated binding mechanisms and could contribute to the rational development of diagnostic probes, with potential applications in early detection, disease progression monitoring, and in vivo imaging of neurodegenerative pathologies.

## Results and Discussion

We first carried out MD simulations of ligand aggregates in aqueous environments. The cluster size distribution analysis showed that, in the dimeric system, the two most populated configurations accounted for 21.3 and 20.3% of the total occurrences, respectively, with the remaining configurations exhibiting a gradual decline in population (Figure 2a). For trimeric clusters, the distribution was more evenly spread, with the three most frequent configurations contributing 12.5, 12.1, and 9.9%, respectively (Figure 2b). A similar trend was observed in tetrameric assemblies, where the most populated cluster comprised 11.7% of the total population, followed by a gradual decline across other configurations (Figure 2c). Pentameric structures, while less frequent, displayed more complex spatial arrangements, with the most populated cluster representing 6.7% of all pentameric states (Figure 2d). As displayed by the representative structures for dimer to pentamer, shown in Figure 2, the π-planar regions of the GTP-1 molecules form π–π stacking interactions and the piperidine rings are oriented in various directions. These results indicate an increase in structural diversity and a more even distribution of populations among different configurations as the oligomer size increases.

**Figure 2:**
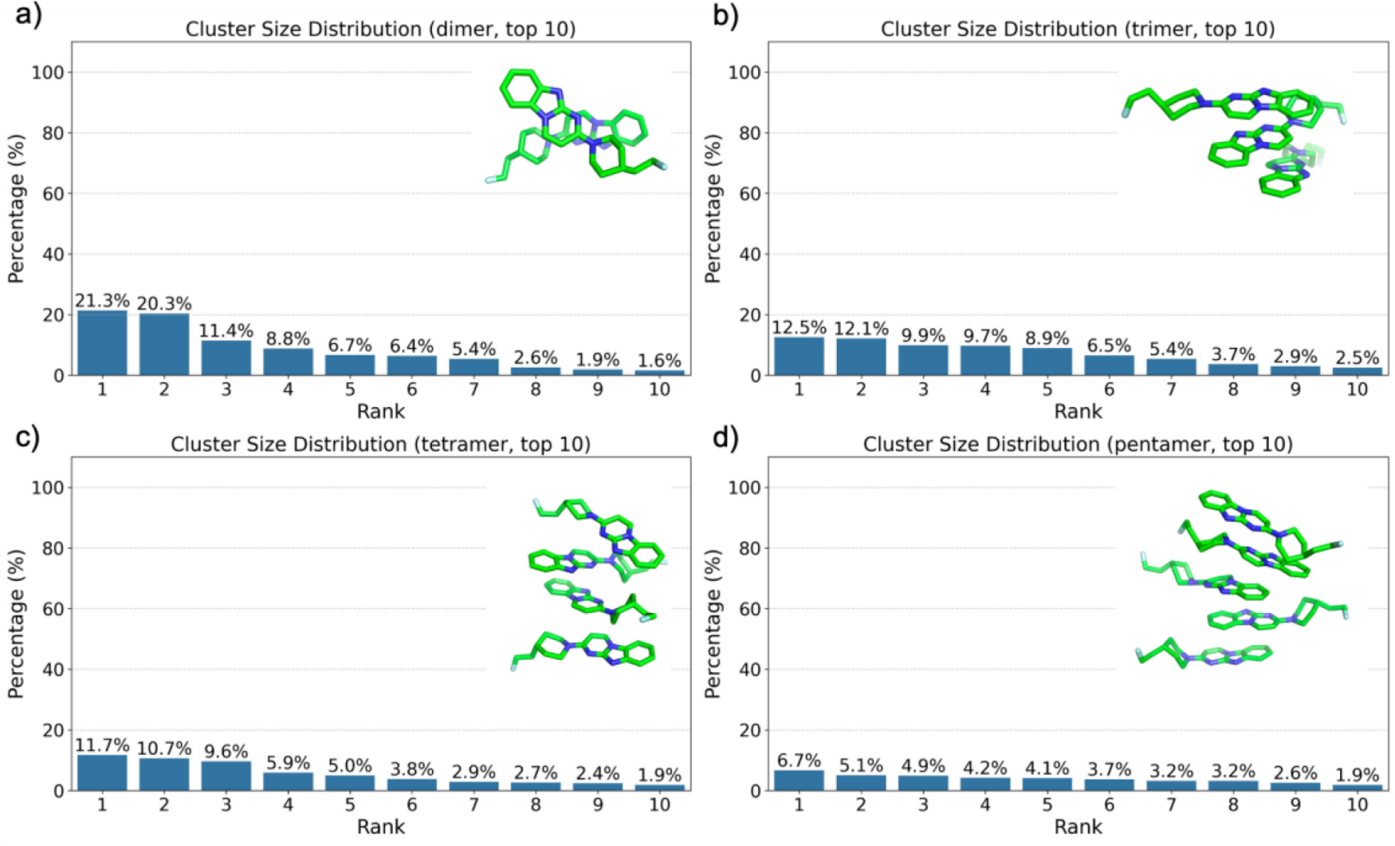
Cluster size distribution of GTP-1 aggregates in aqueous environment for (a) dimer, (b) trimer, (c) tetramer, and (d) pentamer, displaying the top 10 ranked clusters based on their occurrence percentage. Representative molecular structures for the top-ranked clusters are shown alongside each distribution.

The progressive decrease in cluster population percentages from dimers to pentamers, combined with the absence of dominant structural motifs, suggests that GTP-1 aggregation in aqueous environments is primarily driven by nonspecific hydrophobic interactions and a lack of stable configurations (Figure S1). This conclusion is also supported by the structural models shown in Figure 2, which reveal variability in molecular orientations and packing arrangements across all oligomeric states.

In contrast to the solution-phase behavior, GTP-1 aggregation on tau fibril surfaces exhibited highly organized structural arrangements (Figures 3 and S2). Analysis of conformational distributions within discrete oligomeric states (dimers, trimers, tetramers, pentamers) demonstrated characteristic structural arrangements, with higher-order aggregates exhibiting reduced conformational flexibility. On the tau fibril surface, dimeric assemblies displayed a strong preference for specific configurations, with the most populated cluster comprising 41.3% of the total occurrences and the top three configurations collectively accounting for 80.3% of all observed dimers (Figure 3a). This templating effect became even more pronounced in higher-order oligomers, where a single dominant configuration encompassed 96.6% of trimeric clusters (Figure 3b). In the case of tetrameric and pentameric assemblies, complete conformational specificity was observed, with all structures adopting a single configuration (100%, Figure 3c,d). As shown in the representative structures in Figure 3, both the π-planar region and the piperidine ring of GTP-1 are well aligned from the dimer to the pentamer. These findings suggest that the tau fibril surface imposes strong structural constraints on GTP-1 aggregation, guiding the formation of highly ordered assemblies.

**Figure 3:**
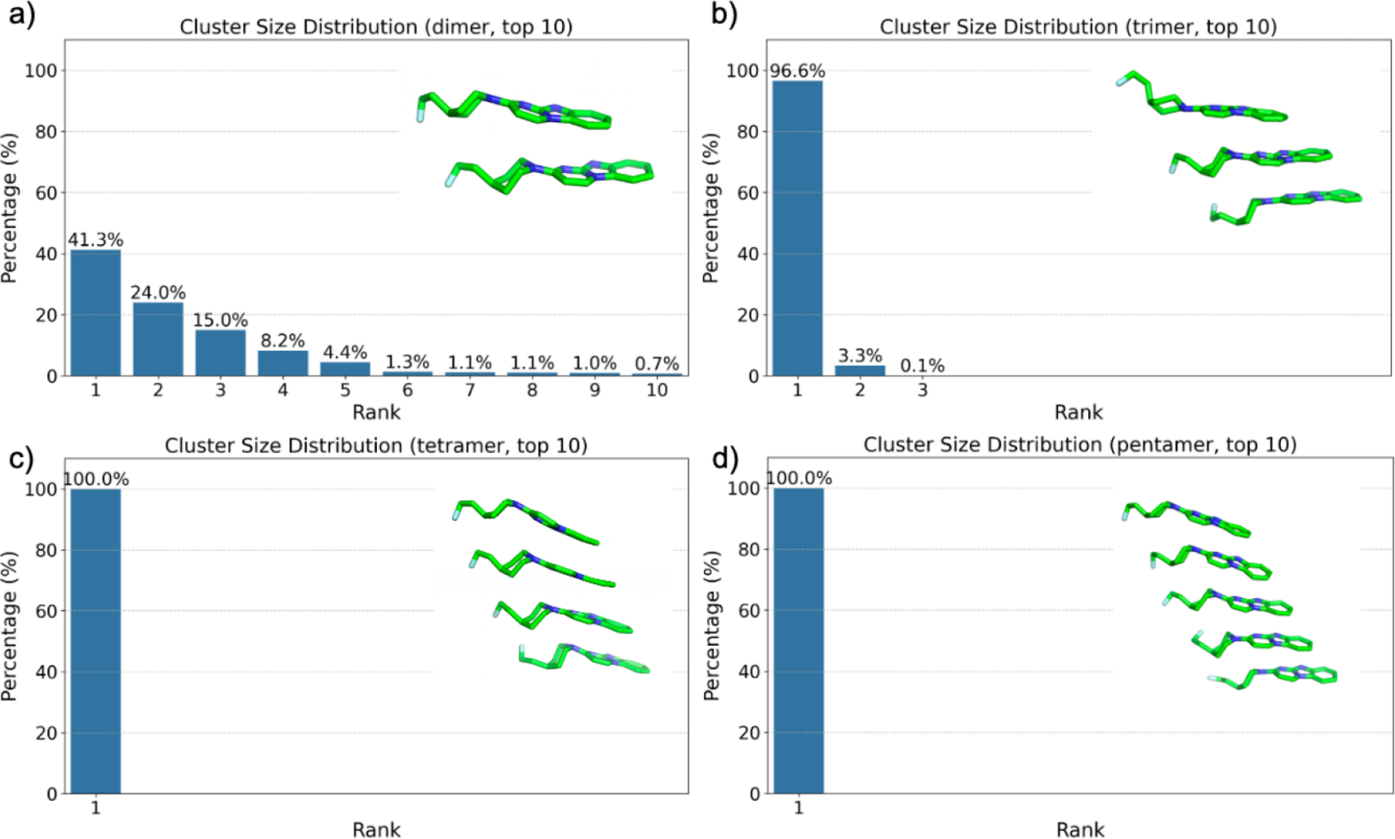
Cluster size distribution of GTP-1 aggregates on fibril surface for (a) dimer, (b) trimer, (c) tetramer, and (d) pentamer, displaying the top 10 ranked clusters based on their occurrence percentage. Representative molecular structures for the top-ranked clusters are shown alongside each distribution.

Structural models illustrate increasingly ordered arrangements with oligomer size, suggesting cooperative interactions between the protein surface and bound ligands. While dimeric structures exhibited clear orientational preferences, they retained some conformational flexibility, as evidenced by the presence of multiple significant clusters. In contrast, trimeric through pentameric assemblies displayed near-complete conformational homogeneity, reflecting highly specific surface recognition and binding modes.

The transition from moderate conformational selection in dimers to nearly absolute specificity in larger oligomers suggests that fibrillar surfaces impose geometric constraints on ligand assembly. These results indicate that amyloid fibril surfaces act as molecular templates, restricting the conformational landscape of bound ligands and stabilizing ligand assemblies in a crystal-like conformation.

The GTP-1 aggregation behavior differs markedly between aqueous solutions and fibril surfaces. In solution, ligand clusters exhibit significant conformational diversity, leading to a broad and heterogeneous distribution of structural arrangements. In contrast, fibril surfaces promote the formation of highly ordered aggregates with strong configurational specificity, particularly in larger oligomers. These findings highlight the critical role of the protein surface in directing ligand organization and demonstrate how environmental context shapes molecular aggregates.

### Thermodynamic Analysis of Fibril-Templated Self-Assembly

Previous simulations of ligand aggregates in solution and on fibrillar surfaces suggest that dimers serve as nucleation points for aggregation, providing both a thermodynamic and structural foundation for the growth of larger assemblies. However, due to limited sampling of ligand aggregation on fibrillar surfaces, a more comprehensive exploration was necessary. To address this, we performed 1000 independent 100 ns simulations of GTP-1 dimers in the fibril surface environment, each initiated from different ligand orientations, to enhance conformational sampling and capture a broader range of aggregation dynamics. To gain deeper insight into the thermodynamic related of fibril-templated aggregation, we analyzed the free energy landscapes of GTP-1 dimer formation based on key intermolecular distances. (Figure 4). In aqueous solution, the free energy landscape revealed two distinct minima corresponding to head-to-head (H_H) and head-to-tail (H_T) configurations. The H_H configuration is characterized by distances between key ligand groups (*d*1 ∼ 7 Å, *d*2 ∼ 5 Å, Figure 4c), representing a compact and stable state. The H_T configuration, with distances of *d*1 ∼ 12 Å and *d*2 ∼ 11 Å, represents another distinct minimum, indicating an alternative stable state with different interaction characteristics.

**Figure 4:**
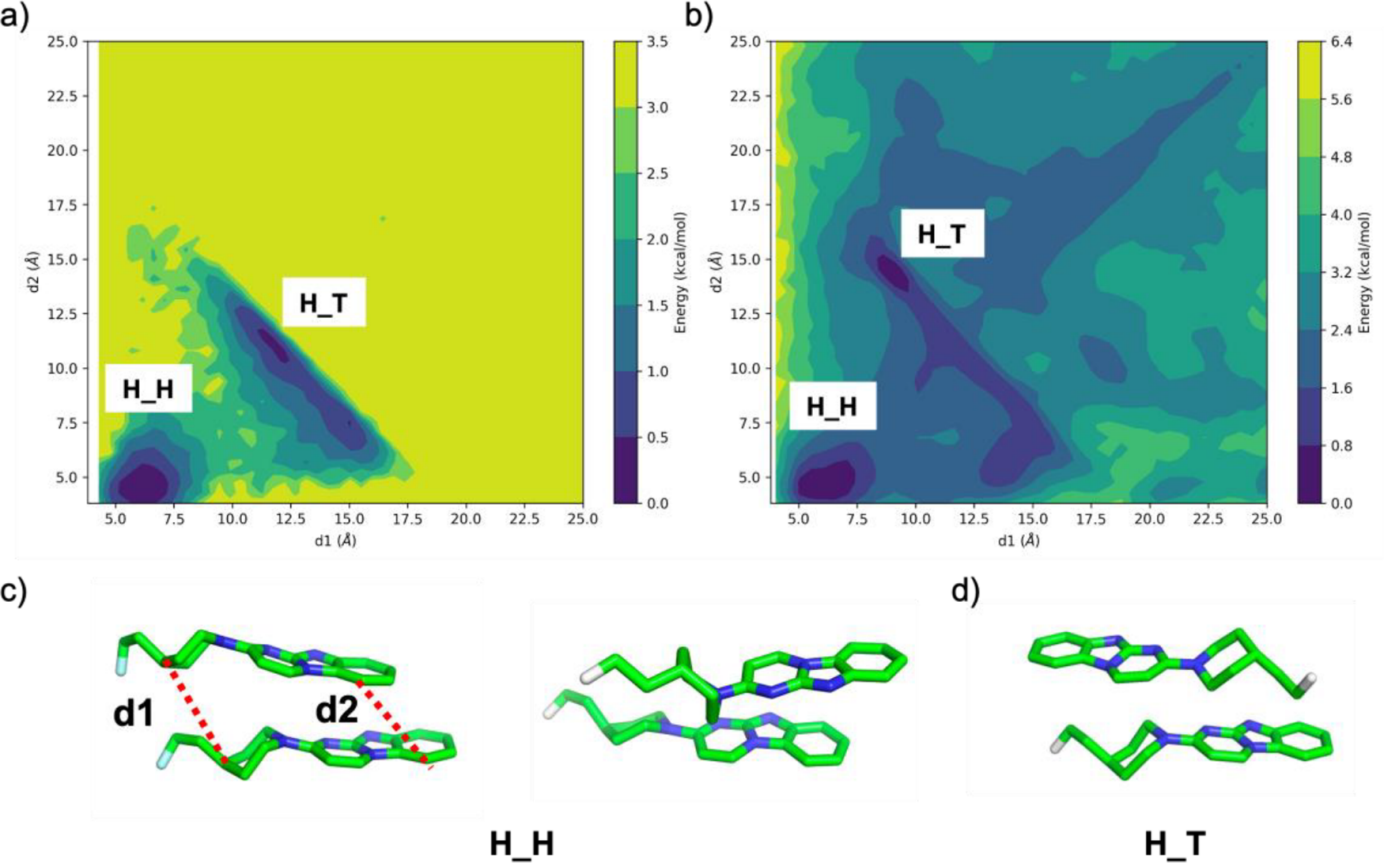
Free energy surfaces illustrating the relative stability of molecular dimer configurations based on distances *d*1 and *d*2 between key structural features. Contour map of the free energy highlighting the two predominant configurations, H_H (head-to-head) and H_T (head-to-tail), characterized by minima in the energy surface for (a) in aqueous and (b) fibril surface environments. Molecular structures for representative configurations are displayed below: (c) H_H configuration and d) H_T configuration. Note that the crystal structure refers to the H_H state.

On the tau fibril surface, the free energy landscape exhibited similarities to the solution phase but also key differences. The H_H configuration remained largely unchanged (*d*1 ∼ 7 Å, *d*2 ∼ 5 Å), suggesting that the fibril surface does not significantly disrupt the interactions stabilizing this state. In contrast, the H_T configuration shifted to a distinct arrangement (*d*1 ∼ 9 Å, *d*2 ∼ 14 Å), indicating that fibril surfaces influence this state by altering its interaction geometry. Changes in the H_T configuration suggest that fibril surfaces introduce specific directional interactions, modulating the energy landscape of bound aggregates. By altering the relative stabilities of key configurations, fibril surfaces impose constraints that guide ligand assembly toward ordered structures less accessible in bulk solution. This analysis provides insight into the mechanistic basis of fibril-templated aggregation and highlights the influence of environments on aggregates.

### Kinetic Characterization of Fibril-Associated Aggregates

Using solvent-accessible surface area (SASA) and ligand RMSD as indicators of aggregation, we classified the trajectories into three categories: on-pathway, off-pathway, and nonaggregated. Both on-pathway and off-pathway trajectories correspond to the formation of aggregates and are characterized by frames with SASA < 250 Å^2^ (Figures S3 and S4). On-pathway trajectories were further defined as those containing frames with ligand RMSD ≤ 2.0 Å, calculated relative to the crystal structure, indicating configurations capable of forming crystal-like assemblies. Off-pathway trajectories, while also aggregated (SASA < 250 Å^2^), lacked frames with ligand RMSD ≤ 2.0 Å relative to the crystal structure, representing noncrystal-like aggregation. In contrast, nonaggregated trajectories were identified by all the frames with SASA > 250 Å^2^, showing no evidence of significant aggregation (Figure S4).

Of the 1,000 trajectories analyzed, 12.6% were classified as on-pathway, 34.6% as off-pathway, and 52.8% as nonaggregated. Analysis of the on-pathway and off-pathway trajectories revealed distinctive kinetic signatures characterizing the fibril-templated aggregation dynamics of GTP-1 (Figure 5). The analysis identified two primary configurational states— H_H and H_T —and examined their interconversion rates. For on-pathway trajectories, which can lead to the formation of crystal-like structures, kinetic analysis revealed highly asymmetric transition probabilities. The forward transition rate from H_H to H_T configurations was notably low (kH_H→H_T = 0.05), while the reverse transition occurred with substantially higher probability (kH_T→H_H = 0.39). This asymmetry suggests the presence of strong energetic barriers that constrain transitions away from crystal-like states. Structural models indicate that these barriers likely arise from optimized protein–ligand contacts that stabilize specific conformations. In contrast, off-pathway trajectories, where no frames with ligand RMSD ≤ 2.0 Å are present, exhibit more balanced transition probabilities between H_H and H_T states (kH_H→H_T = 0.28, kH_T→H_H = 0.25). This near-equilibrium pattern suggests a more uniform energy landscape for off-pathway configurations, where the absence of specific stabilizing interactions allows for easier conformational changes. Structural representations show that while these configurations maintain basic ligand–ligand contacts, they lack the precise geometric alignment characteristic of crystal-like structures.

**Figure 5:**
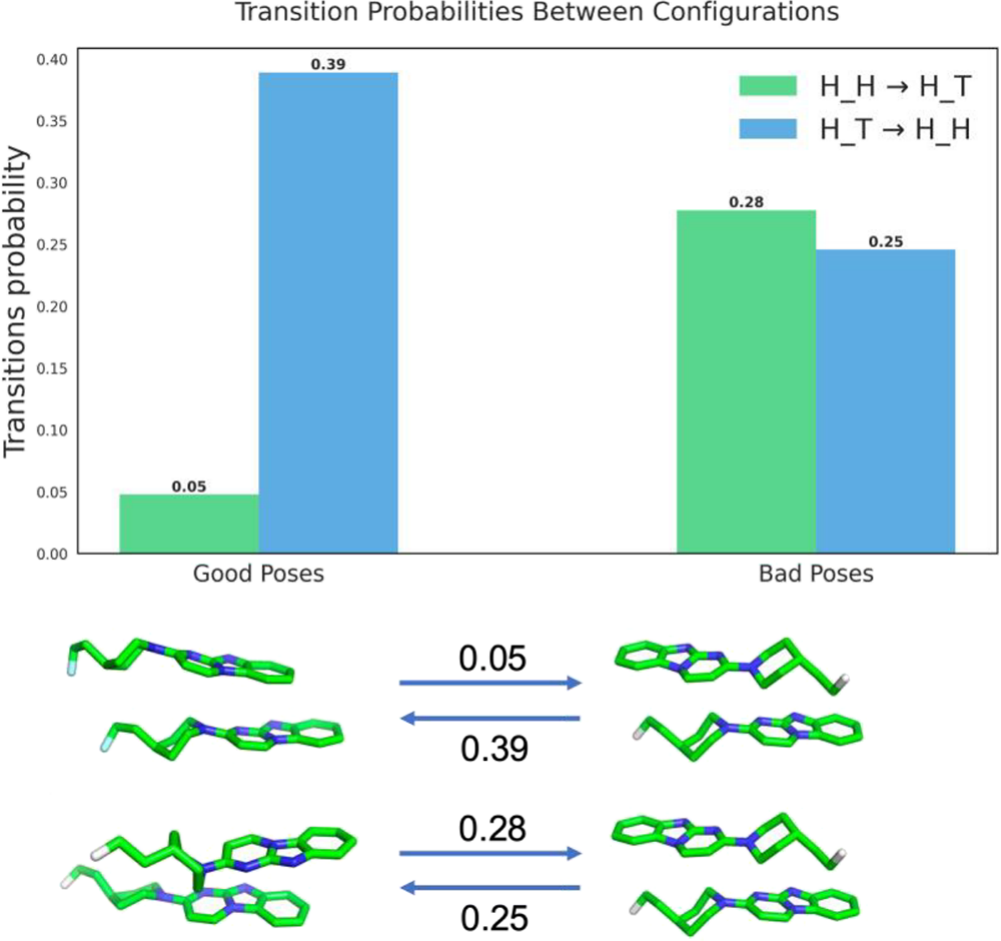
Transition probabilities between molecular dimer configurations. The bar chart quantifies the probabilities of transitions from H_H to H_T and H_T to H_H configurations under two categories: Good Poses (on-pathway) and Bad Poses (off-pathway). Representative molecular structures for each configuration and the corresponding transitions are illustrated below.

These findings suggest a hierarchical aggregation process in which the initial ligand binding establishes key protein contacts, templating the formation of ordered aggregates through cooperative ligand–ligand and ligand-protein interactions. Once a crystal-like structure is formed, the system becomes trapped in this configuration due to strong energetic barriers and optimized interactions. These barriers stabilize the aggregate in a deep energy well, making transitions to alternative configurations both energetically unfavorable and kinetically improbable. This trapping effect not only ensures the persistence of the ordered state but also reinforces the assembly process, promoting the continued formation of highly ordered structures. Additionally, it can be anticipated that longer simulation times would increase the likelihood of forming on-pathway trajectories due to the kinetic trapping effect. This expectation is supported by our analysis of the 1000 trajectories: when examining only the first 20 ns, 7% of the trajectories belonged to the on-pathway category, whereas this proportion increased to 12.6% when analyzing 100 ns.

Overall, these results provide key insight into the molecular mechanisms underlying fibril-templated ligand aggregation, emphasizing the thermodynamic and kinetic factors that govern molecular recognition in protein–ligand systems. The clear distinction between the kinetics of on-pathway and off-pathway trajectories underscores the critical role of specific protein–ligand interactions in guiding the formation of ordered molecular aggregates.

### Structural Basis of Fibril-Mediated Stabilization

To better understand the forces stabilizing GTP-1 aggregates on fibrillar surfaces, we analyzed the molecular interactions involved, identifying hydrogen bonding as a critical determinant of aggregate stability and specificity (Figure 6). Quantitative assessment of hydrogen bonds revealed significant differences between on-pathway and off-pathway trajectories, providing mechanistic insights into templated assembly.

**Figure 6:**
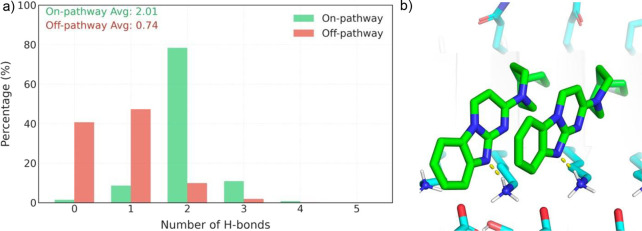
Distribution of hydrogen bonds (H-bonds) in on-pathway and off-pathway trajectories. (a) Histogram showing the percentage distribution of the number of H-bonds. (b) Representative molecular configuration highlighting H-bonds (yellow dashed lines) in the crystal structure.

On-pathway trajectories displayed highly specific hydrogen bonding patterns, with a mean of 2.01 intermolecular hydrogen bonds per structure. Over 80% of conformations maintained exactly two hydrogen bonds, forming a sharp distribution. These hydrogen bonds were exclusively formed between the protein surface and the ligand, anchoring the ligand in defined orientations. This alignment facilitated additional stabilization through ligand–ligand π–π interactions, creating a cooperative network that reinforced the extended H_H configuration. These precise and cooperative interactions highlight the role of hydrogen bonds as molecular anchors that position ligands on the fibril surface to promote ordered assembly.

In contrast, off-pathway aggregates exhibited reduced hydrogen bonding, with a mean of 0.74 intermolecular hydrogen bonds. These hydrogen bonds were inconsistent and unable to anchor ligands in defined orientations. While ligand–ligand π–π stacking interactions were present, they could not compensate for the absence of stable hydrogen bonds, preventing the formation of well-ordered aggregates. The broader and bimodal distribution of hydrogen bonds in off-pathway aggregates—where approximately 40% of structures formed no hydrogen bonds and 45% formed only a single bond—underscores their lack of cooperative stabilization. The majority of hydrogen bonds are formed between the N6 atom of GTP-1 and the side chain of residue K353 on the Tau protein (Figures 6b and S5). These findings suggested that in the studied system, hydrogen bonding plays a key role for the fibril-templated ligand aggregation. Therefore, a rewarding strategy is to design ligands that incorporate hydrogen bond donors and acceptors in optimal positions, which could enhance the binding affinity and specificity of diagnostic and therapeutic molecules targeting amyloid structures.

For the ligands that form stacked binding mode, two components contribute to the binding free energy: protein–ligand binding and ligand–ligand stacking. To quantify the protein–ligand and ligand–ligand interactions, we performed MM/GBSA analysis. The binding free energy is calculated based on the following formula:

$$\Delta G_{\mathrm{binding}}=n\Delta G_{\mathrm{pro}-\mathrm{lig}}+(n-1{)}^{*}\Delta G_{\mathrm{lig}-\mathrm{lig}}$$ 1

where *n* represents the number of monomers in the system, Δ*G*_pro–lig_ is the protein–ligand interaction energy, and Δ*G*_lig–lig_ is the ligand–ligand interaction energy.

The ligand–ligand interaction energy, Δ*G*_lig–lig_, is calculated using the following formula:

$$\Delta G_{\mathrm{lig}-\mathrm{lig}}=\Delta G_{\mathrm{pro}-{\mathrm{lig}}_{\dim\mathrm{er}}}-\Delta G_{\mathrm{pro}-{\mathrm{lig}}_{1}}-\Delta G_{\mathrm{pro}-{\mathrm{lig}}_{2}}$$ 2

where Δ*G*_pro–lig_dimer__ is the protein–ligand interaction energy where the dimer is treated as a single entity in the calculation. Δ*G*_pro–lig_1__ and Δ*G*_pro–lig_2__ represent the protein–ligand interaction energies between the protein and each of the two ligands in the dimer, respectively. In the practical application, two dimer systems are generated from the trimer trajectory, and the average binding free energy is calculated therefrom.

As shown in Table 1, Δ*G*_pro–lig_ is predicted to −18.18 ± 1.89 kcal/mol and Δ*G*_lig–lig_ to −2.87 ± 0.10 kcal/mol. These results indicate that protein–ligand interactions play crucial roles in the fibril-templated ligand aggregation process.

**Table 1:** Energy Component for Ligand–Fibril Interactions

energy component	Δ*G*_pro–lig_ (kcal/mol)	Δ*G*_lig–lig_ (kcal/mol)
values	–18.18 ± 1.89	–2.87 ± 0.10

### General Features of Fibril-Templated Aggregation

Templated-Assisted Self-Assembly (TASA) is a mechanism widely recognized in supramolecular systems, particularly in materials chemistry and nanomaterials, where templates actively regulate assembly dynamics, driving specificity and stability in the resulting structures.^22–25^ A defining feature of TASA is its ability to promote cooperativity in assembly. Initial binding events establish precise interactions between the template and components, which subsequently nucleate further binding through the formation of an extended network of interactions.^20^ This cooperative process is often amplified by the geometric and chemical properties of the template surface, actively influencing the size, orientation, and alignment of the assembling domains.^26^ Furthermore, TASA systems often exhibit accelerated kinetics compared to spontaneous aggregation, with template surfaces reducing the entropic cost of assembly and facilitating rapid nucleation and propagation steps. This allows ordered aggregates to form even at low component concentrations, where spontaneous assembly in solution is typically not observed.^17^

In this study, we identified similar features in the fibril-templated aggregation of GTP-1, suggesting that this process can be classified within the framework of TASA. Fibril surfaces stabilized ligand orientations and configurations, facilitating ordered aggregation that would otherwise be energetically unfavorable in solution. Cooperative hydrogen bonding between the protein surface and ligands acted as molecular anchors, precisely aligning ligands and enabling the formation of extended aggregates through π–π stacking interactions. If GTP-1 aggregation on tau fibril surfaces also adheres to the principles of TASA, it suggests that GTP-1 could undergo aggregation on tau surfaces even at low concentrations, a hallmark of TASA systems. This provides a potential explanation for a key debate in PET studies, which argues that stacked binding cannot occur in vivo due to the significantly lower ligand concentrations compared to those used in cryo-EM studies.^11^ Our findings suggest that stacked binding might still be possible even at low concentrations due to the existence of fibril templates. However, further experimental validation is required to confirm this mechanism in the context of protein-mediated systems.

## Conclusions

This study provides insight into the molecular mechanisms by which pathological fibril surfaces template the aggregation of small molecule ligands. Using extensive molecular dynamics simulations (∼100 μs) of the model ligand GTP-1 in both solution and fibril-bound states, we demonstrate that protein surfaces actively promote ordered ligand aggregates. This occurs by restricting conformational flexibility and introducing specific stabilizing interactions, such as protein–ligand hydrogen bonds and ligand–ligand π–π stacking interactions.

Free energy landscape and kinetic analyses revealed the dominant role of extended configurations in fibril-associated aggregates formed through on-pathway trajectories, which are distinct from the disordered assemblies observed in off-pathway trajectories. Detailed structural investigations further emphasized the cooperative nature of fibril-templated aggregation, driven by precise protein–ligand hydrogen bonding and ligand–ligand interaction networks. These findings establish fibril-templated assisted aggregation as a conceptual framework for understanding protein-mediated ligand aggregation. In this framework, protein templates not only facilitate aggregation under otherwise unfavorable in aqueous conditions but also actively regulate the aggregation process through cooperative interactions and kinetic acceleration.

Collectively, these findings highlight the key molecular principles governing fibril-mediated ligand aggregation and provide a foundation for designing small molecule probes and therapeutics targeting pathological fibrils. By harnessing protein-templated aggregation, this work offers new avenues for developing diagnostic agents in neurodegenerative diseases.

## Methods

### Computational Methods

Molecular dynamics simulations were performed using AMBER22.^27^ The cryo-EM structure (PDB ID: 8FUG) was initially processed using the Schrödinger Protein Preparation Wizard. Translational and rotational matrices were calculated using a protocol reported previously,^11^ and applied to generate a system comprising 10 monomers. Three distinct simulation systems were prepared: (1) cryo-EM systems with different number of ligands (from dimer to pentamer) in their experimentally determined positions with the presence of a tau fibril, (2) ligand-only systems (from dimer to pentamer) in water using the crystallographic conformations, and (3) a dimer formation simulation where one ligand maintained its crystallographic conformation while the second ligand was positioned with its center of mass 1–2 nm away in randomized orientations. For (3), the initial conformations for the second ligand were generated according to the following steps:

starting from the first ligand,

1. A random rotation matrix is generated using Euler angles. The ligand is then rotated around its center of mass using this matrix to ensure diverse orientations.
2. A random translation vector is applied to shift the center of mass of the ligand within a 1–2 nm radius from the original position. This helps to randomize the spatial distribution of the ligands around the given site.
3. The generated conformation is subjected to steric clash detection. Only keep the conformation without steric clash with the first ligand and tau fibril structure.
4. After 1–3 steps, one conformation of the second ligand is generated. 1–3 steps are repeated until 1000 spatially diverse conformations are generated.

Ligand partial atomic charges were derived using the AM1-BCC charge model.^28^ The system was parametrized using the ff14SB force field for proteins and GAFF2 for ligands within the tleap module.^29^ Each complex was solvated in an octahedral box of TIP3P water with a 10 Å buffer distance, and counterions were added to achieve charge neutrality. Energy minimization was conducted for 50,000 steps using the steepest descent algorithm to eliminate unfavorable contacts. The systems were then gradually heated from 0 to 300 K over 100 ps under NVT conditions, with protein and ligand heavy atoms restrained using a force constant of 10 kcal/mol·Å^2^. Subsequently, a 100 ps NPT equilibration was performed at 300 K and 1 atm using the Berendsen barostat with a 2 ps relaxation time.^30^

Production MD simulations were conducted in the NPT ensemble for 100 ns. The SHAKE algorithm was employed to constrain bonds involving hydrogen atoms, allowing a 2 fs integration time step.^31^ Long-range electrostatic interactions were treated using the Particle Mesh Ewald method with a 10 Å cutoff.^32^

### Cluster Analysis

Conformational analysis was performed through hierarchical clustering of the ligand trajectories. Prior to clustering, all frames were aligned to the first ligand. A pairwise RMSD matrix was constructed based on the atomic coordinates of ligand heavy atoms. Hierarchical clustering was implemented using the centroid linkage method with a distance criterion threshold of 40.0 Å. The clustering procedure generated distinct conformational families, with each cluster represented by its centroid structure. The resulting clusters were analyzed for population distribution and structural characteristics.

### Heatmap Analysis

We employed two key metrics: (1) native contacts, which measure structural similarity to the crystal reference state (values closer to 1 indicate greater similarity to the native state), and (2) coordination number, which characterizes the degree of ligand aggregation (higher values indicate more extensive aggregation).

Native contacts are defined relative to the crystal structure of the reference state. For each corresponding atom pair (atom i in ligand 1 and the equivalent atom i in ligand 2), the distance in the crystal structure serves as the reference value. A native contact is considered formed if the current distance between atoms falls within ± 0.2 nm of this reference distance. A native contact is considered formed if the current distance between atoms falls within ± 0.2 nm of this reference distance. All the native contacts are then summed and then normalized to a maximum value of 1.0.

For coordination number quantification, we used the following equation:

$$\underset{i\in A}{\sum }\underset{i\in B}{\sum }s_{ij}$$ 3

where *s*_*ij*_ is determined according to the following equation:

$$s_{ij}=\frac{1-{\left(\frac{r_{ij}-d_{0}}{r_{0}}\right)}^{n}}{1-{\left(\frac{r_{ij}-d_{0}}{r_{0}}\right)}^{m}}$$ 4

In this equation, *r*_*ij*_ represents the distance between atoms *i* and *j*, where *i* belongs to ligand A and *j* belongs to ligand B. We used parameters *n* = 6, *m* = 8, *d*_0_ = 0, and *r*_0_ = 0.45 nm.

For oligomeric structures (trimer to pentamer), we employed a specialized calculation approach to account for multiple pairwise interactions. Taking the trimer system as an example, we first calculated all pairwise native contacts between ligands. To represent the overall structural similarity to the reference state while focusing on the most favorable interactions, we selected the two highest native contact values, summed them, and then divided by 2 (as a trimer contains two key interactions). This normalization ensures that native contact values remain comparable across different oligomeric states, with values consistently ranging from 0 to 1.

The same selection strategy was applied to coordination number calculations, where we considered the strongest pairwise interactions to characterize the degree of aggregation in higher-order oligomers. However, for coordination numbers, we summed the selected highest values without averaging, as more extensive ligand aggregation naturally results in higher coordination numbers.

### Thermodynamic Analysis

The free energy landscape was characterized using two reaction coordinates: the head-to-head distance (H_H) and head-to-tail distance (H_T) between ligand pairs. Two-dimensional probability distributions *P*(H_H, H_T) were computed from the trajectory data. The free energy surface was calculated according to

$$\Delta G(H\text{\_}H,\;H\text{\_}T)=-RT\ln\frac{P(H\text{\_}H,\;H\text{\_}T)}{P_{0}}$$ 5

where *R* is the gas constant, *T* is the temperature (300 K), and *P*_0_ represents the highest probability state observed in the distribution.

### Kinetics Analysis

The dimer formation process was analyzed across 1000 independent trajectories. Aggregation states were classified based on the solvent accessible surface area (SASA), with trajectories exhibiting SASA values below 250 Å^2^ designated as aggregated states. These aggregated trajectories were further categorized through RMSD analysis relative to the cryo-EM conformation. Trajectories containing frames with ligand RMSD values <2 Å were classified as on-pathway, while those maintaining RMSD values >2 Å were designated as off-pathway. The conformational transitions between head-to-head and head-to-tail arrangements were quantified through probability analysis of the respective geometric parameters.

### MM/GBSA

MM/GBSA calculations were executed using AMBERTools.^33^ The analysis employed the modified GB model (igb = 2) with a physiological salt concentration of 0.150 M. The free energy analysis was performed on the terminal 20 ns segment of 100 ns trajectories, focusing exclusively on the three central ligands within the pentameric assembly. The total binding free energy was decomposed into Δ*G*_pro–lig_ and Δ*G*_lig–lig_, components, corresponding to protein–ligand and ligand–ligand interaction energies, respectively.

## Supplementary Material



## Notes

Figures S1–S2, heatmap analysis of ligand aggregation in different environments; Figures S3–S4, time evolution of SASA of cryo-EM and two other representative structures; and Figure S5, hydrogen-bond formation analysis (PDF)
