# Supplementary material for: Molecular Insight into Amyloid Fibril-Templated Aggregation of Biomarkers
Source: ACS Chem Neurosci. 2025 May 27;16(11):2076–84. doi: 10.1021/acschemneuro.5c00103 (PMC12142574; doi:10.1021/acschemneuro.5c00103)
Supplement: Supplementary file 1 [file cn5c00103_si_001.pdf]

Supporting Information

For

Molecular Insight into Amyloid Fibril-Templated Aggregation of Biomarkers

Rongfeng Zou,[a*] Hans Ågren[a,b*]

[a]Department of Physics and Astronomy, Uppsala University, Box 516, SE-751 20 Uppsala, Sweden
[b]Faculty of Chemistry, Wroclaw University of Science and Technology, Wyb. Wyspianskiego 27, PL-50370 Wroclaw, Poland

Correspondence:
Rongfeng Zou, rongfeng.zou@physics.uu.se
Hans Ågren, hans.agren@physics.uu.se

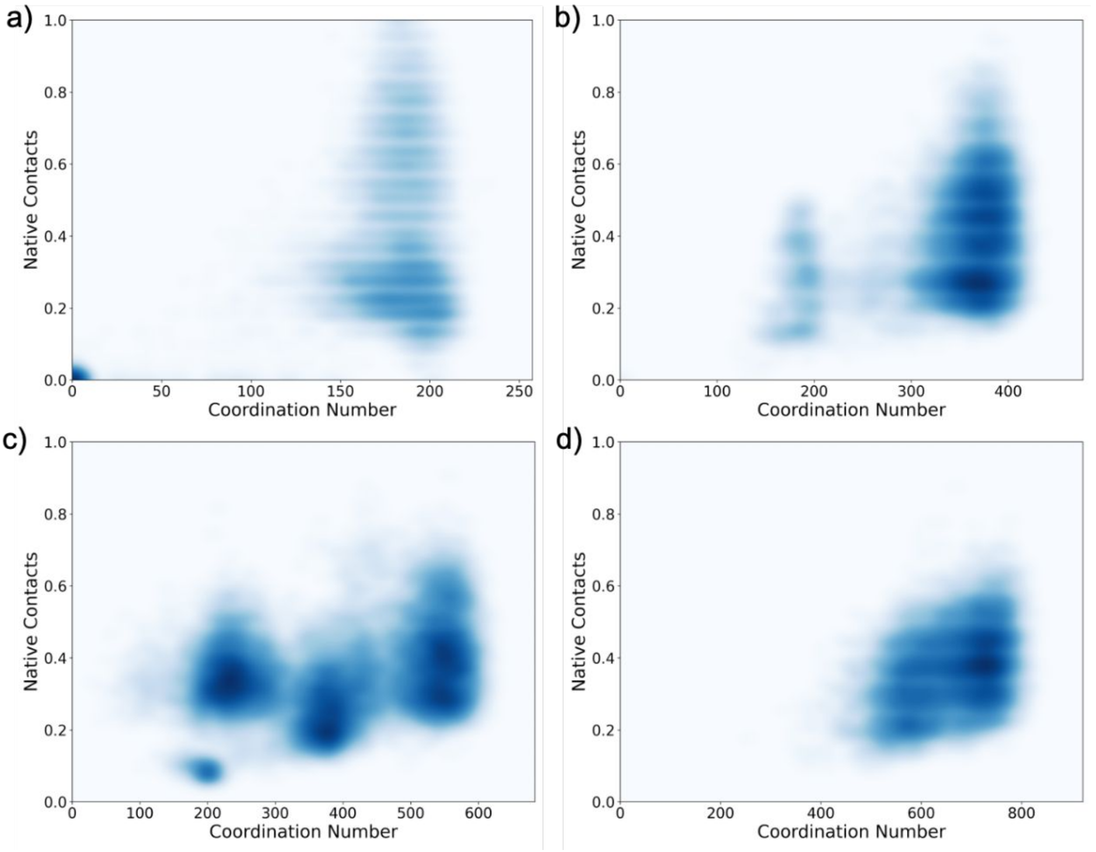

Figure S1. Heatmap analysis of ligand aggregation in aqueous environment showing the relationship between coordination number (x-axis) and native contacts (y-axis) for different oligomeric states. Darker blue intensity indicates higher probability densities. (a) Dimeric structures. (b) Trimeric structures. (c) Tetrameric structures. (d) Pentameric structures. Coordination number quantifies the extent of molecular aggregation, while native contacts measure structural similarity to the crystal reference state. In aqueous environment, GTP-1 aggregates exhibit low native contact values regardless of coordination number, indicating non-specific interactions.

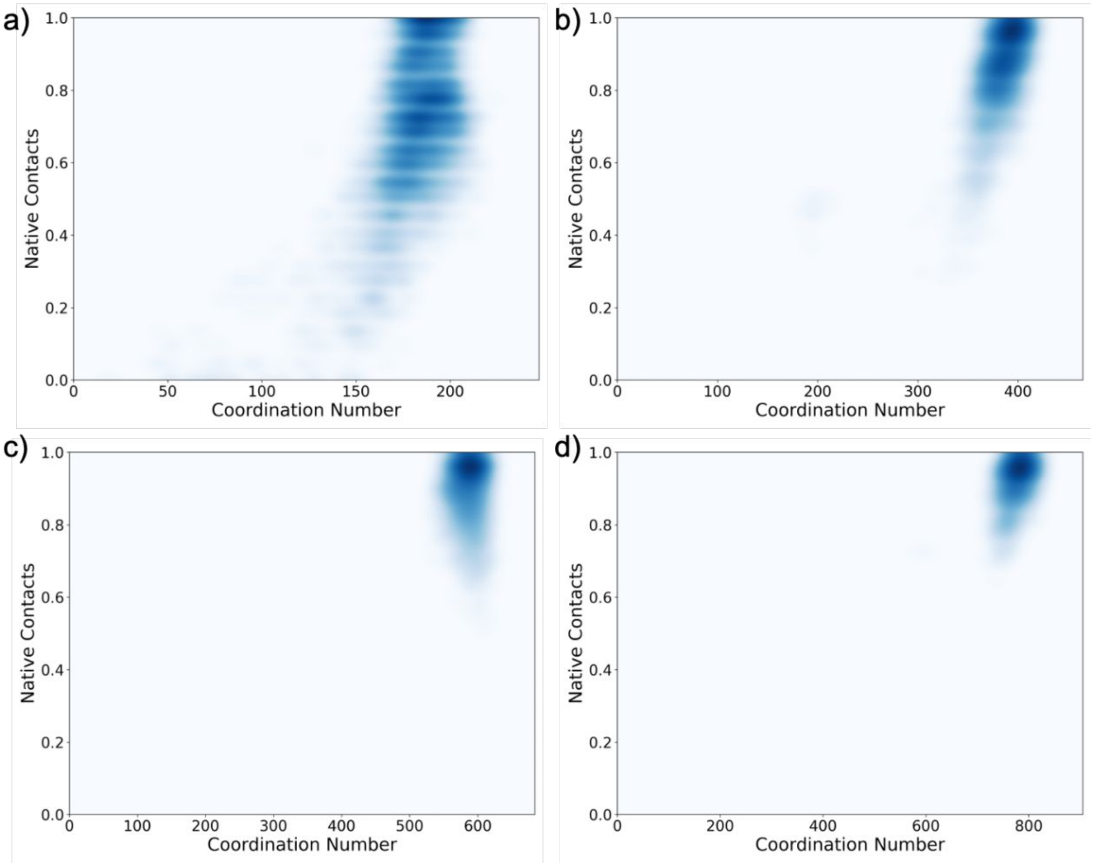

Figure S2. Heatmap analysis of ligand aggregation on the tau fibril showing the relationship between coordination number (x-axis) and native contacts (y-axis) for different oligomeric states. Darker blue intensity indicates higher probability densities. (a) Dimeric structures. (b) Trimeric structures. (c) Tetrameric structures. (d) Pentameric structures. Coordination number quantifies the extent of molecular aggregation, while native contacts measure structural similarity to the crystal reference state. Higher coordination numbers strongly correlate with higher native contact values (0.8-1.0), demonstrating that GTP-1 molecules preferentially adopt conformations closely resembling the crystal structure conformation on the tau fibril surface.

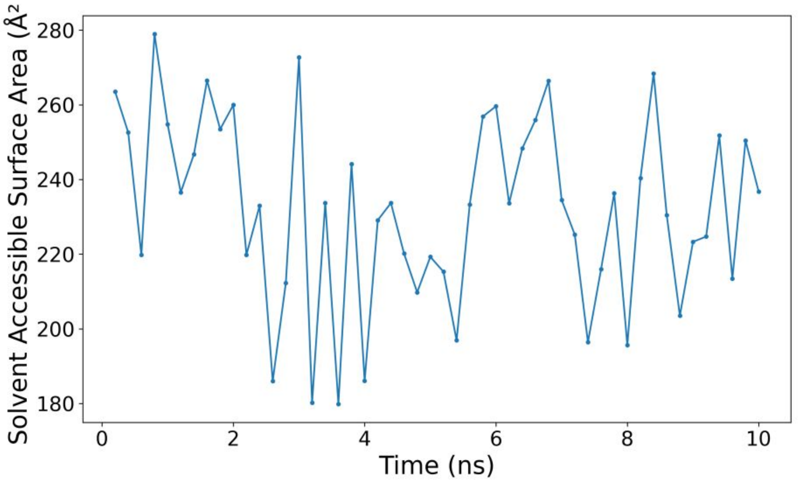

Figure S3. Time evolution of SASA using the cryo-EM structure as the starting structure. The average SASA is around 250 Å² in the first 2 ns of the simulation, Therefore, 250 Å² is used as the criterion for identifying aggregated states.

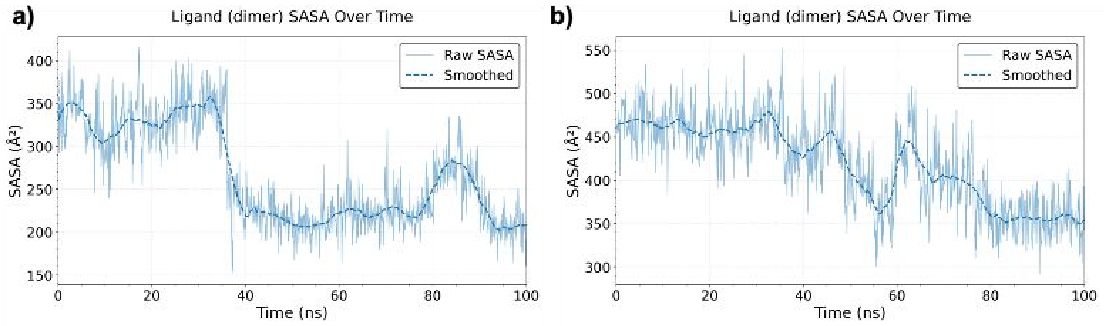

Figure S4. Time evolution of ligand dimer SASA for two representative molecular dynamics trajectories: (a) forming and (b) not forming aggregates. Raw SASA measurements (blue) and smoothed data (black dashed line) were monitored over 100 ns. A SASA value of 250 Å² was utilized as the threshold for aggregation classification.

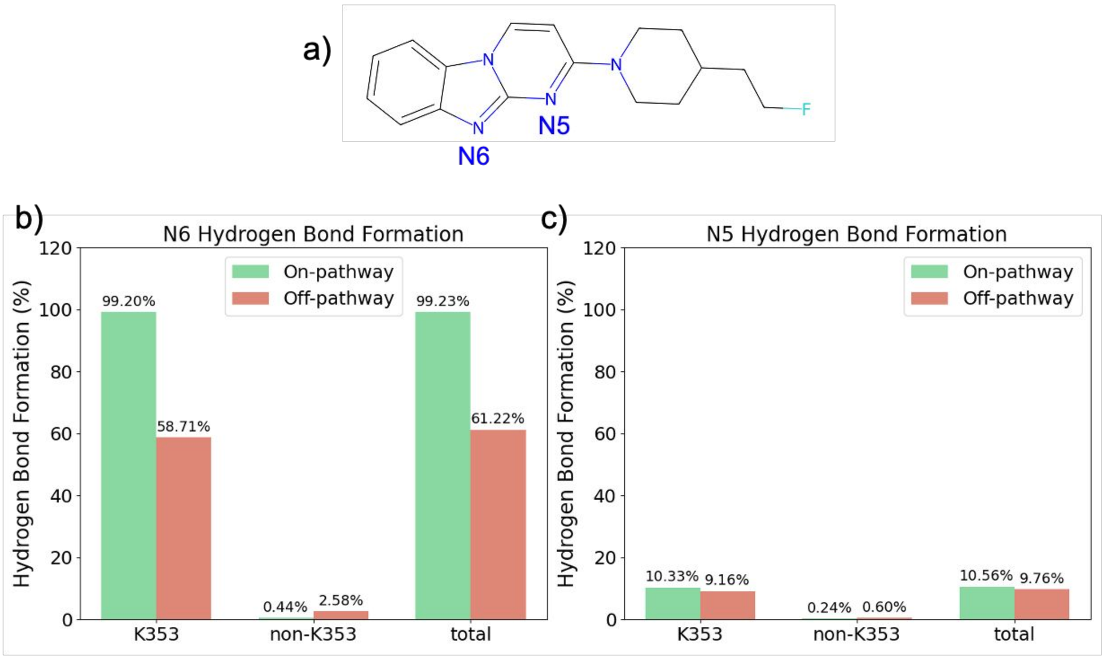

Figure S5: a) Chemical structure of GTP-1 with N5 and N6 labeled as hydrogen bond acceptors. Hydrogen bond formation percentages at b) N6 and c) N5 positions for on-pathway (green) and off-pathway (red) trajectories. X-axis shows K353, non-K353 interaction types, and total hydrogen bond formation.
